# The gut-bone marrow axis in acute leukemia: an evidence-graded review of microbiota-immune crosstalk and therapeutic implications

**DOI:** 10.3389/fcimb.2026.1926235

**Published:** 2026-09-02

**Authors:** Xinfu Zou, Jinyang Bu, Aojiao Chu, Zheng Zhang, Qin Zheng, Meihong Luo

**Affiliations:** Baoshan Hospital, Shanghai University of Traditional Chinese Medicine, Shanghai, China

**Keywords:** acute leukemia, gut dysbiosis, gut-bone marrow axis, microbial metabolites, microbiota-immune crosstalk

## Abstract

The gut microbiota is increasingly associated with immune dysfunction, treatment-related complications, and clinical outcomes in acute leukemia (AL), but the literature remains dominated by observational cohorts and mechanistic extrapolation from non-leukemia settings. This review critically evaluates gut dysbiosis, microbial metabolites, immune-cell remodeling, and microbiota-directed interventions in AL through an evidence-graded, leukemia-centered framework. We distinguish AL-specific human associations, AL-specific experimental evidence, mechanistic evidence from other disease contexts, and hypotheses that remain to be tested. The available data support a context-dependent gut-bone marrow model in which dysbiosis may influence AL through three non-exclusive routes: loss of barrier-supportive microbial functions with increased translocation of microbial products; systemic distribution of microbial metabolites and inflammatory mediators that act on bone marrow stromal, myeloid, and lymphoid compartments; and trafficking of microbiota-educated immune cells from the intestine to distant tissues. Among candidate mediators, AL-specific experimental evidence is strongest for reduced butyrate availability, intestinal barrier injury, lipopolysaccharide leakage, and accelerated leukemia progression in murine AML. By contrast, direct microbiota-derived metabolite-induced transcriptional reprogramming of human leukemia blasts, including whether butyrate regulates the same targets in blasts as in colonocytes or mature immune cells, has not been established. We further show that apparently conflicting microbial signatures can be explained by differences in disease subtype, age, treatment phase, antibiotic exposure, diet, sampling, analytical platform, and functional redundancy among taxa. On this basis, we propose a two-hit model and a set of testable hypotheses for paired stool-plasma-bone marrow studies, metabolite tracing, immune-cell clonotype tracking, and mechanism-based intervention trials. This evidence-graded gut-bone marrow perspective defines the distinct contribution of the review and provides a more conservative basis for interpreting microbiota-targeted strategies in AL.

## 1 Introduction

Acute leukemia (AL) comprises malignant clonal disorders arising from hematopoietic stem or progenitor cells and is broadly classified as acute myeloid leukemia (AML) or acute lymphoblastic leukemia (ALL) (Xiao et al., 2024). Its pathological hallmarks include the accumulation of immature hematopoietic cells in bone marrow and peripheral blood, suppression of normal hematopoiesis, and tissue infiltration (Weinberg, 2024). Leukemia remains an important cause of cancer mortality worldwide, while AL contributes substantially to this burden (Bray et al., 2024; Rafiq et al., 2024). Clinically, AL commonly presents with anemia, bleeding, infection, and organ infiltration (Ni et al., 2025). Despite advances in chemotherapy, targeted therapy, immunotherapy, and allogeneic hematopoietic stem cell transplantation (allo-HSCT), marked heterogeneity in response and major complications, including severe infection and graft-versus-host disease (GVHD), continue to limit durable benefit. These observations justify investigation of host and microenvironmental factors that may modify disease biology and treatment tolerance.

In addition to leukemia-cell-intrinsic genetic and epigenetic abnormalities, immune dysregulation, chronic inflammation, barrier injury, and metabolic remodeling are associated with AL progression and treatment outcomes (Wiertsema et al., 2021; Afzaal et al., 2022; O'Riordan et al., 2025). The intestine contains extensive gut-associated lymphoid tissue, and the gut microbiota contributes to mucosal barrier maintenance, colonization resistance, and production of bioactive metabolites, including short-chain fatty acids (SCFAs), bile acid derivatives, and tryptophan metabolites (Smith and Garrett, 2011; Rowland et al., 2018; Zheng et al., 2020; Trepka et al., 2025). Dysbiosis may deplete protective functions, expand pathobionts, alter metabolite availability, and facilitate microbial-product translocation; however, the strength and direction of these effects depend on host context (Maciel-Fiuza et al., 2023; Wong and Yu, 2023; Robles-Vera et al., 2025). In AL, chemotherapy, broad-spectrum antibiotics, neutropenia, mucosal injury, and allo-HSCT are major ecological perturbations, making it difficult to separate disease-associated dysbiosis from treatment-induced dysbiosis (Rashidi et al., 2022; Zhou et al., 2022; Pötgens et al., 2023; Guevara-Ramírez et al., 2024; Tian et al., 2025).

**Figure 1:**
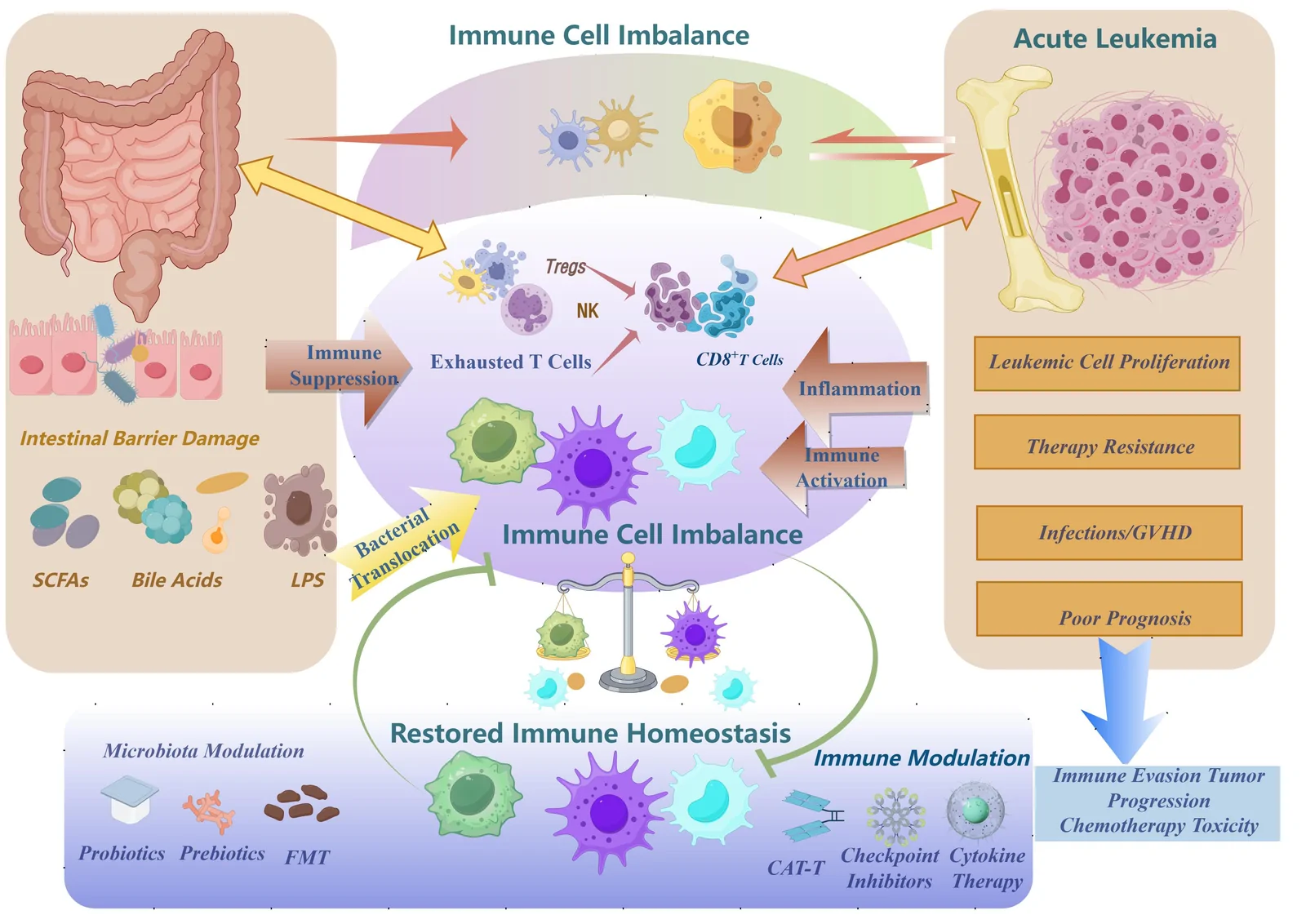
Conceptual framework of the gut-bone marrow axis in acute leukemia. Gut dysbiosis and intestinal barrier injury may alter the availability of microbial metabolites and permit translocation of microbial products such as lipopolysaccharide. Candidate systemic routes include circulating metabolites and cytokines, microbial products, and trafficking of microbiota-educated immune cells. These signals may act on bone marrow stromal cells, hematopoietic progenitors, dendritic cells, T cells, B cells, macrophages, natural killer cells, and potentially leukemia blasts. The figure should be interpreted as an evidence-graded model: several links are supported by non-leukemia or transplantation studies, whereas direct demonstration in human AL remains limited. AL, acute leukemia; SCFAs, short-chain fatty acids; LPS, lipopolysaccharide; DCs, dendritic cells; NK, natural killer cells; GVHD, graft-versus-host disease; FMT, fecal microbiota transplantation. Created using BioRender.com.

To avoid conflating association with causation, this review uses an explicit evidence hierarchy. Evidence is described as: (i) AL-specific human evidence when derived from patients with AML or ALL; (ii) AL-specific experimental evidence when a causal pathway has been tested in leukemia models; (iii) contextual mechanistic evidence when the pathway has been demonstrated in non-leukemia immune, intestinal, or hematopoietic models; and (iv) hypothesis when the proposed connection has not been directly tested. This distinction is particularly important because many widely cited microbiota-immune mechanisms were established in inflammatory bowel disease, solid tumors, infection, or transplantation rather than in untreated AL. The distinct contribution of this review is therefore not a further catalogue of taxa or treatment associations. Instead, we organize the field around a leukemia-centered gut-bone marrow axis, examine how soluble metabolites, translocated microbial products, and microbiota-educated immune cells might reach and remodel the marrow niche, and use this framework to explain discrepancies and generate testable hypotheses. **Figure 1** provides the overall conceptual map. Subsequent sections critically synthesize dysbiosis, immune-cell abnormalities, treatment-related perturbations, and the opportunities and limitations of dietary, fecal microbiota transplantation (FMT), probiotic, and prebiotic strategies.

## 2 Gut microbiota dysbiosis and leukemia

Across published cohorts, recurrent findings include reduced microbial richness or diversity, depletion of obligate anaerobes and butyrate-producing taxa, and expansion of facultative pathogens such as Enterobacteriaceae or *Enterococcus*. Nevertheless, no single taxonomic pattern is universal. Newly diagnosed untreated AML cohorts (Wang et al., 2022; Pötgens et al., 2024), pediatric ALL cohorts sampled before or during chemotherapy (Chua et al., 2020; Gao et al., 2020; Liu et al., 2020; Xu et al., 2023), recovered AL or high-risk myelodysplastic syndrome outpatients (Vázquez et al., 2024), chemotherapy-exposed AML cohorts (Rattanathammethee et al., 2020), and murine leukemia models (Wang et al., 2022; Wu et al., 2023) represent biologically different settings. Representative studies and their comparators are summarized in **Table 1**. The apparent contradictions across studies are therefore informative rather than merely technical noise. Age affects baseline microbiome maturation and immune tone; AML and ALL differ in lineage, treatment, and host age distribution; treatment stage changes nutritional intake, mucosal integrity, and antimicrobial exposure; and 16S rRNA sequencing, shotgun metagenomics, and metabolomics measure different layers of the ecosystem. In addition, taxonomic differences do not necessarily imply functional differences because unrelated taxa can perform overlapping metabolic functions, while strain-level variation can produce different effects within the same genus. These factors can explain why a taxon reported as depleted in one cohort may be unchanged or enriched in another. Accordingly, current human data support dysbiosis as a context-dependent ecological state associated with AL rather than a stable disease-specific signature. The strongest AL-specific experimental chain is provided by the AML study linking depletion of butyrate-producing bacteria to barrier injury, systemic LPS exposure, and accelerated leukemia progression in mice (Wang et al., 2022). Even this study does not establish that the same pathway operates in all AML subtypes or in human ALL. Other reported taxa should therefore be treated as cohort-specific associations unless they are replicated across centers and connected to a defined metabolite, host receptor, immune-cell phenotype, and clinical endpoint.

**Table 1:** Summary of studies reporting gut microbiota compositional alterations in acute leukemia.

Study	Study population/comparator	Study design/model	Reported gut microbiota alterations
Wang et al., 2022	Newly diagnosed, untreated AML patients and healthy controls; AML mice models were also used.	Clinical cohort combined with experimental AML models.	Compared with healthy controls, AML patients showed lower gut microbiota diversity. At the phylum level, *Firmicutes* was decreased and *Bacteroidota* was increased in AML. At the genus level, *Bacteroides* was enriched, whereas *Faecalibacterium*, *Roseburia*, *Subdoligranulum*, and *Bifidobacterium* were decreased in AML patients.
Gao et al., 2020	Children with ALL and healthy controls; pre-treatment ALL patients and remission-stage ALL patients were also compared.	Pediatric case-control study based on 16S rDNA sequencing.	Lower OTU counts and lower overallmicrobial abundance than in healthy controls; no clear alpha-or beta-diversity differences between remissionstage ALL and healthy controls;Enrichment of *Acinetobacter*, *Actinomyces*, *Bosea*, *Brevundimonas*, *Enterococcus*, *Megasphaera*, *Oribacterium*, *Rhizobium*, *Ruminiclostridium, Sphingomonas*, *Tyzzerella*, *Veillonella*, *Anaerostipes, Bifidobacterium*, *Blautia*, *Collinsella*, *Dialister*, *Dorea, Erysipelatoclostridium*, *Faecalibacterium*, *Lactobacillus, Oscillibacter*, *Prevotella*, *Roseburia, Ruminococcus*, *and Terrisporobacter* in AL patients compared with the control group
Vázquez et al., 2024	Outpatients recovered from AL or high-risk myelodysplastic syndrome and healthy controls.	Cross-sectional metagenomic study.	Compared with healthy controls, recovered AL/high-risk MDS outpatients showed higher abundance of *Enterobacteriaceae*, *Prevotella*ceae, *Rikenellaceae*, *Alistipes*, and *Prevotella*, and lower abundance of *Bacteroidaceae, Bifidobacteriaceae, Lachnospiraceae*, *Bacteroides*, *Bifidobacterium*, and *Blautia*. No significant difference in overall microbial diversity was reported between groups.
Chua et al., 2020	Children with ALL compared with age- and ethnicity-matched healthy children; samples were collected before chemotherapy, during chemotherapy, and after cessation of chemotherapy.	Longitudinal pediatric clinical study using 16S rRNA sequencing.	Before chemotherapy, children with ALL showed greater inter-individual variability and enrichment of Bacteroidetes and *Bacteroides* compared with healthy controls. *Bacteroides* abundance decreased after chemotherapy, and microbiota composition became closer to that of healthy controls after treatment cessation, although beta diversity remained distinct.
Xu et al., 2023	Pediatric ALL patients and healthy controls.	Pediatric case-control microbiota study.	Significant beta-diversity differences were observed between childhood ALL patients and healthy controls. Compared with the control group, the ALL patients showed increased abundances of *Edwardsiella tarda* and *Prevotella maculosa* and were associated with IL-10 levels.
Wu et al., 2023	Adult patients with AML/AML mouse model and healthy controls.	A combined approach integrating clinical observation and animal models.	Compared with the control group, the AML mouse model showed increased abundances of *Bacteroidaceae*, *Marinifilaceae*, *Bacteroidales_uncultured*, and *Rikenellaceae*, while the abundances of *Atopobiaceae*, *Campylobacteraceae*, *Coriobacteriales Incertae Sedis*, *Peptococcaceae*, *Erysipelotrichaceae*, *WCHB1–41 Other*, *Eggerthellaceae*, and Pasteurellaceae were decreased; Compared with healthy controls, AML patients showed a decreased abundance of *Firmicutes*.
Pötgens et al., 2024	Adult patients with AML and controls.	Clinical gut microbiota profiling study.	Compared with the control group, AML patients showed increased abundances of *Blautia* and *Chloroflexi*, and a decreased abundance of *Tenericutes*.
Yu D. et al., 2021	ML patients and healthy controls.	Case-control microbiome and metabolome study.	Compared with healthy controls, patients with ML showed increased abundances of *Actinobacteria*, *Acidobacteria*, *Chloroflexi*, *Sphingomonas*, *Lysobacter*, *Helicobacter*, *Lactobacillus*, *Enterococcus*, and *Clostridium sensu stricto 1*, whereas the abundance of Streptococcus was decreased.
Rattanathammethee et al., 2020	AML patients receiving chemotherapy and AML patients not receiving chemotherapy.	Cross-species metabolome and microbiome analysis.	Compared with AML patients not receiving chemotherapy, AML patients receiving chemotherapy showed a decreased abundance of Firmicutes and an increased abundance of Bacteroidetes.
Liu et al., 2020	Children with ALL and healthy controls.	Multicenter, cross-sectional, prospective clinical study using shotgun metagenomics and metabolomics.	ALL patients differences beta-diversity versus healthy controls; Compared with healthy controls, children with ALL showed decreased relative abundances of Edwardsiella tarda and Prevotella maculosa.

## 3 Immune cells and acute leukemia

AL is accompanied by remodeling of the bone marrow immune microenvironment, but the direction of causality is often difficult to establish. Human studies describe reduced NK-cell abundance or maturation, altered activating and inhibitory receptors, exhausted CD8+ T-cell phenotypes, Th1/Th17/Treg imbalance, abnormal B-cell regulation, M2-like macrophage polarization, and quantitative or functional defects in dendritic cells (Boutilier and Elsawa, 2021; Sendker et al., 2021; Zhang et al., 2021; Bednarski et al., 2022; Lv et al., 2022; Tettamanti et al., 2022; Basak et al., 2023; Cao et al., 2023; Hope et al., 2023; Jia et al., 2023; Koller et al., 2023; Palomares et al., 2024; Perzolli et al., 2024; Wang CY. et al., 2024; Upadhyay et al., 2025). These abnormalities are associated with immune escape, treatment resistance, and relapse, and may create a permissive niche for leukemia persistence; however, most data do not show that any single immune-cell defect initiates AL.

A more useful interpretation is that leukemia cells, treatment exposures, infection, and systemic inflammatory signals interact to produce a dynamic immune network rather than an isolated defect in one cell type. NK-cell dysfunction can weaken cytotoxic surveillance; exhausted or suppressive T-cell states can limit adaptive clearance; macrophages and regulatory B-cell subsets can reinforce inhibitory cytokine and checkpoint signaling; and defective dendritic-cell maturation can reduce antigen presentation. These compartments are also plausible downstream targets of gut-derived signals. The key mechanistic question is therefore not simply whether each cell type is abnormal in AL, but whether microbiota-derived metabolites, microbial products, or gut-educated immune cells reach the marrow and measurably modify these abnormalities.

## 4 Interactions between the gut microbiota and immune cells

The gut microbiota regulates immunity through microbe-associated molecular patterns and microbially derived metabolites (Iyer et al., 2023; Wan et al., 2023; Kim et al., 2024). SCFAs, LPS, bile acid derivatives, and tryptophan metabolites can influence antigen presentation, lineage differentiation, cytokine secretion, and barrier maintenance. Importantly, most mechanistic evidence summarized in Sections 4.1-4.5 derives from non-leukemia models. Unless AL-specific evidence is explicitly stated, these pathways should be interpreted as biologically plausible mechanisms rather than demonstrated causes of immune remodeling in AL.

### 4.1 Dendritic cells

In experimental intestinal and immune models, SCFAs can alter dendritic-cell (DC) maturation through HDAC inhibition, G-protein-coupled receptor signaling, and changes in cytoskeletal or metabolic programs (Nastasi et al., 2015; Yao et al., 2022; Filardy et al., 2023; Inamoto et al., 2023; Hays et al., 2024; Kim et al., 2024). Butyrate and propionate have reduced LPS-induced CD40, IL-6, and IL-12p40 in bone marrow-derived DCs, while GPR109A signaling in colonic antigen-presenting cells favors IL-10, retinaldehyde dehydrogenase activity, and Treg induction (Nastasi et al., 2015; Hays et al., 2024). Bile acid-TGR5-cAMP signaling can similarly generate DCs with lower IL-12 and TNF-alpha production (Su et al., 2023; Lee et al., 2024). These data show that microbial metabolites can tune DC activation, but they do not establish the same response in leukemia-associated DCs or marrow DC progenitors. In AL, paired measurements of fecal metabolites, plasma exposure, marrow DC phenotype, and antigen-presenting function are lacking; thus, the proposed microbiota-DC-leukemia pathway remains an extrapolation.

### 4.2 T cells

Mechanistic support for microbiota-T-cell regulation is strong in non-leukemia systems. *Bacteroides fragilis* polysaccharide A and selected *Clostridioides* strains can expand Foxp3+ Treg populations, whereas segmented filamentous bacteria promote Th17 differentiation (Fabersani et al., 2021; Zegarra-Ruiz et al., 2021; Grenda et al., 2022; Al-Fakhrany and Elekhnawy, 2024). SCFAs can influence NF-kappaB signaling, HDAC activity, cellular metabolism, and the balance among Treg, Th17, effector, and memory T-cell states (Facchin et al., 2024; Yu et al., 2024; Chen et al., 2025). These effects are not uniformly anti-inflammatory or antitumor: the direction depends on metabolite concentration, receptor expression, activation state, nutrient availability, and tissue context. In AL, butyrate depletion and T-cell abnormalities coexist (An et al., 2025; Kong and Li, 2025; Nowicka and Gil, 2025), but a complete causal chain from a defined microbial function to systemic metabolite exposure, marrow T-cell reprogramming, and leukemia control has not been demonstrated. The microbiota-metabolite-T-cell model should therefore be treated as a testable mechanism rather than an established feature of AL.

### 4.3 B cells

Microbiota-B-cell interactions are best established in mucosal humoral immunity. IgA shapes the abundance and localization of commensal organisms, while microbial signals and SCFAs influence B-cell metabolism, plasma-cell differentiation, and IgA production (Honda and Littman, 2016; Bunker et al., 2017; Upadhyay and Littman, 2022; Kawamoto et al., 2023; Kawamoto and Hara, 2024; Mann et al., 2024; Takeuchi et al., 2024). In AL, chemotherapy, antibiotic exposure, mucosal injury, and depletion of normal B-cell compartments may disrupt this reciprocal control and thereby weaken colonization resistance. However, direct evidence connecting a specific microbial metabolite to marrow B-cell dysfunction, regulatory B-cell activity, or leukemia outcome is minimal. The most defensible current hypothesis is indirect: loss of mucosal IgA and barrier integrity may increase microbial translocation and systemic inflammation, which could then influence the marrow niche.

### 4.4 Macrophages

Macrophages provide a mechanistically plausible bridge between barrier injury and marrow inflammation. In non-leukemia models, *Clostridium butyricum* can induce intestinal IL-10+ macrophages through TLR2/MyD88 signaling, while butyrate can restrain NLRP3 activation, promote oxidative metabolism, and enhance repair-associated phenotypes (Scott et al., 2018; Gasaly et al., 2021; Shao et al., 2021; van der Lelie et al., 2021; Zhang Z. et al., 2022; Lu et al., 2024; Meng et al., 2024; Paz Del Socorro et al., 2024). Conversely, translocated LPS activates TLR4-NF-kappaB signaling and can promote inflammatory macrophage programs (Zhou and Li, 2023). AL-specific experimental evidence is more limited but important: in the AML model reported by Wang et al., barrier injury increased systemic LPS exposure and LPS accelerated leukemia progression, whereas butyrate improved barrier integrity and reduced LPS leakage (Wang et al., 2022). That study supports a barrier-LPS-leukemia pathway, but it did not trace gut-derived LPS to a defined bone marrow macrophage subset or establish that macrophage polarization mediated the leukemia phenotype. Direct lineage tracing and macrophage-specific receptor perturbation are therefore needed.

### 4.5 Natural killer cells

NK-cell responses to SCFAs are strongly context dependent. In one non-leukemia model, reduced butyrate impaired an IL-18-dependent pathway required for tissue-resident NK-cell maturation, whereas other *in vitro* studies found that butyrate suppressed activation receptors and inflammatory cytokine production in human peripheral-blood NK cells (Tian et al., 2023; Zaiatz-Bittencourt et al., 2023; Pérez et al., 2024; Shang et al., 2024). Additional studies suggest that SCFAs can enhance selected antitumor NK-cell functions (Pérez et al., 2024). These apparently opposing findings likely reflect differences in NK-cell subset, tissue location, cytokine stimulation, exposure duration, and metabolite concentration. No study has yet demonstrated that a gut-derived SCFA reaches the marrow at a sufficient concentration to restore anti-leukemia NK-cell function in patients with AL. Accordingly, microbiota-NK interactions are promising but remain mechanistically unvalidated in leukemia.

## 5 A leukemia-centered gut-bone marrow axis: integrated mechanistic model

### 5.1 Candidate routes from the intestine to the bone marrow

The anatomical distance between the intestine and bone marrow requires a transport mechanism. Three non-exclusive routes are supported to different degrees. More broadly, commensal microbiota have been shown to promote hematopoiesis and innate immune readiness during bacterial infection in non-leukemia models (Khosravi et al., 2014). First, soluble metabolites and host mediators can enter portal and systemic circulation. In non-leukemia models, microbiota-derived lactate reaches the marrow and activates GPR81-dependent stem cell factor production by LepR+ stromal cells, while microbiota-dependent butyrate availability regulates macrophage-mediated iron recycling and stress hematopoiesis (Lee et al., 2021; Zhang D. et al., 2022). These studies establish that microbial metabolites can act on marrow niche cells, but they do not demonstrate equivalent effects in AL. Second, intestinal barrier disruption can permit systemic exposure to MAMPs, bacterial DNA, or viable organisms. In AML mice, reduced butyrate, barrier injury, and LPS leakage were linked to faster leukemia progression (Wang et al., 2022). In a separate non-leukemia model, microbial translocation after barrier disruption induced Mincle-dependent training of myeloid progenitors in the bone marrow (Robles-Vera et al., 2025). Together, these findings support a route in which microbial products alter marrow inflammatory tone, but the responsible receptors and target cells in human AL remain undefined. Third, immune cells educated in the intestine may traffic to the marrow. Direct evidence exists outside leukemia: intestinal TNF+ T cells and Th17 cells migrated to bone marrow through S1PR1-, CXCR3-, and CCL20-dependent pathways in an ovariectomy model (Yu M. et al., 2021). This demonstrates biological feasibility, not an established AL mechanism. In leukemia, the relevant experiment would require paired gut, blood, and marrow sampling with T-cell receptor clonotype or cellular barcoding to show that microbiota-responsive clones leave the intestine, enter the circulation, and accumulate in marrow.

### 5.2 Leukemia-centered interpretation of microbial metabolites

Butyrate illustrates why tissue context matters. In colonocytes, butyrate is an energy substrate and supports epithelial barrier integrity; in mature immune cells, it can signal through GPCRs and inhibit HDACs. The AL-specific study by Wang et al. supports an indirect barrier-mediated mechanism in which reduced microbial butyrate permits LPS leakage (Wang et al., 2022). By contrast, older pharmacologic studies showed that millimolar sodium butyrate or butyrate derivatives could induce histone acetylation, differentiation, growth arrest, or apoptosis in selected AML blasts, particularly core-binding-factor AML (Gozzini et al., 2003). These experiments used drug-like exposures and cannot be assumed to reflect the lower, fluctuating concentrations of microbiota-derived butyrate reaching human marrow. Thus, the same molecule may have distinct intestinal, immune-cell, stromal, and blast-intrinsic actions depending on dose and cellular genotype. For bile acid derivatives and tryptophan metabolites, most receptor-level mechanisms have been established in intestinal or systemic immune cells rather than leukemia blasts. It remains unknown whether AL blasts consistently express functional TGR5, FXR, aryl hydrocarbon receptor, or relevant metabolite transporters at levels that permit direct regulation by microbiota-derived ligands. Likewise, reported amino-acid or metabolite signatures in AML and ALL do not automatically establish microbial origin because leukemia-cell metabolism, diet, hepatic metabolism, renal function, and treatment can generate the same circulating metabolite changes. A leukemia-specific mechanism therefore requires demonstration of microbial production, systemic delivery, target-cell exposure, receptor engagement, and phenotypic rescue.

### 5.3 A context-dependent two-hit model

We propose a two-hit model to integrate the literature. The first hit is ecological and intestinal: AL, diet, antibiotics, chemotherapy, and mucosal injury reduce functional redundancy, deplete barrier-supportive metabolites, or expand pathobionts. The second hit is marrow susceptibility: leukemia genotype, disease burden, stromal remodeling, immune exhaustion, and treatment stage determine how the marrow responds to circulating metabolites, MAMPs, cytokines, or gut-derived immune cells. Neither hit alone is expected to produce a uniform microbiome signature. This model explains why similar taxa can be associated with different outcomes across cohorts and why the same metabolite can be protective in the intestine but suppressive or cytotoxic in another cell compartment. The model also separates three clinically distinct questions that are often conflated: whether dysbiosis contributes to leukemia biology; whether it predicts treatment-related infection or toxicity; and whether restoring the microbiota improves a clinical endpoint. A taxon may be useful for infection prediction without influencing leukemia progression, while an intervention may restore diversity without changing remission or survival. Each claim therefore requires a matched endpoint and an appropriate comparator.

### 5.4 Testable hypotheses

This framework generates four testable hypotheses. First, the barrier-LPS hypothesis predicts that loss of butyrate-producing function precedes increased plasma LPS or bacterial DNA and is followed by a defined marrow myeloid-inflammatory program. Second, the metabolite-niche hypothesis predicts that circulating microbial metabolites correlate with receptor-specific transcriptional changes in stromal cells or hematopoietic progenitors and can be reproduced or blocked in organoid or xenograft models. Third, the immune-trafficking hypothesis predicts shared clonotypes or barcoded gut-educated T cells in blood and marrow, with migration dependent on specific chemokine axes. Fourth, the genotype-context hypothesis predicts that leukemia subtypes differ in metabolite receptor expression and therefore show different blast-intrinsic responses. Paired longitudinal stool, plasma, peripheral blood, and bone marrow samples collected before antibiotics, during induction, at remission, and at relapse would allow these hypotheses to be tested.

## 6 Interactions between the gut microbiota and therapeutic approaches for acute leukemia

### 6.1 Chemotherapy

Chemotherapy is a major ecological perturbation in AL, but baseline disease-associated dysbiosis and treatment-induced dysbiosis should be analyzed separately. Children with ALL and adults with AML may already differ from healthy controls before treatment (Hakim et al., 2018; Pötgens et al., 2024; Vázquez et al., 2024), whereas induction therapy commonly produces abrupt losses of diversity, depletion of obligate anaerobes, expansion of facultative pathogens, and barrier injury (Sun et al., 2018; Rajagopala et al., 2020; Nie et al., 2021; Pötgens et al., 2023; Luo et al., 2024; Wang H. et al., 2024). Longitudinal sampling is therefore more informative than a single post-treatment comparison. Across AML cohorts, higher baseline diversity has been associated with lower subsequent infection risk, while carbapenem exposure, *Enterococcus* domination, or expansion of specific pathobionts has been associated with infectious complications (Galloway-Peña et al., 2020; Zhang et al., 2025; Alvaro et al., 2026). Pediatric ALL studies similarly link baseline or on-treatment microbial features to febrile neutropenia, diarrhea, pneumonia, or delayed neutrophil recovery (Hakim et al., 2018; Nearing et al., 2019; Liu et al., 2021; Wang H. et al., 2024; Sørum et al., 2025). These findings support risk stratification, but antibiotic indication, disease severity, diet, mucositis, and hospitalization are major confounders. Microbiota restoration with MaaT033 after intensive therapy has shown ecological recovery and acceptable safety in AML, but evidence for improved remission, survival, or other hard endpoints remains insufficient (Malard et al., 2025). The most defensible conclusion is that the microbiota is a candidate modifier and biomarker of treatment tolerance and infection risk, not yet a proven determinant of chemotherapy efficacy.

### 6.2 Immunotherapy

In AL, microbiome-specific evidence for immunotherapy is concentrated in CD19 CAR-T studies that include ALL together with lymphoma, rather than in leukemia-only cohorts. Broad-spectrum antibiotic exposure before CAR-T infusion has been associated with poorer survival and greater toxicity, while enrichment of Ruminococcus, *Bacteroides*, or *Faecalibacterium* has been associated with higher response rates (Smith et al., 2022). These associations are biologically plausible because microbiota-dependent metabolites and inflammation can alter T-cell fitness, but confounding by infection severity and treatment intensity cannot be excluded. Oral vancomycin exposure has also been associated with greater CAR-T expansion in B-ALL (Uribe-Herranz et al., 2023), illustrating that microbiota perturbation is not uniformly detrimental. Evidence for blinatumomab, inotuzumab ozogamicin, and checkpoint inhibition remains sparse and largely inferential (Marrapodi et al., 2025). Future studies should distinguish effects on CAR-T expansion, persistence, cytokine release syndrome, neurotoxicity, infection, and antileukemia response rather than treating ‘immunotherapy outcome’ as a single endpoint.

### 6.3 Allogeneic hematopoietic stem cell transplantation

Allo-HSCT provides the strongest clinical evidence connecting gut ecology with outcomes, although many cohorts include multiple hematologic malignancies rather than AL alone. Lower peri-transplant diversity and Enterococcus domination are repeatedly associated with mortality, acute GVHD, and impaired immune reconstitution (Ingham et al., 2019; Peled et al., 2020; Sofi et al., 2021; Rashidi et al., 2023; van Lier et al., 2023; Fujimoto et al., 2024; Yang et al., 2024). Experimental models provide partial causal support by linking SCFAs, IL-22, Treg responses, and barrier integrity to GVHD severity (Sofi et al., 2021). Nevertheless, diversity is a composite ecological measure rather than a mechanism, and associations may reflect antibiotic exposure, conditioning intensity, nutrition, mucositis, and early complications. The possibility that microbiota modulation alters graft-versus-leukemia activity is especially important but remains less well established than the relationship with GVHD and infection. Conclusions should therefore distinguish transplant-related evidence from leukemia-specific disease-control evidence.

## 7 Clinical prospects of gut microbiota-targeted interventions

The gut microbiome is modifiable, but ecological change is not equivalent to clinical benefit. In AL, any microbiota-directed intervention must be evaluated against the competing risks of infection, mucosal injury, drug interaction, and delayed definitive therapy. The appropriate translational sequence is target identification, mechanistic validation, safety assessment, ecological endpoint confirmation, and finally testing of a prespecified clinical endpoint. Current evidence for diet, FMT, probiotics, and prebiotics is therefore discussed as emerging supportive care rather than established antileukemia therapy.

### 7.1 Dietary intervention

Diet can alter substrate availability and microbial metabolism, but evidence specific to microbiota-mediated effects in AL is limited. Observational and animal studies link obesity, hypertriglyceridemia, and high-fat feeding to leukemia burden through pathways such as PPAR-alpha/FLT3, JAK/STAT signaling, inflammatory cytokines, and FABP4-dependent epigenetic regulation (Yan et al., 2017; Hermetet et al., 2020; Kincaid et al., 2022; Wu et al., 2022). These findings demonstrate host metabolic effects but do not prove that the microbiota is the mediator. Maternal dietary associations with childhood AL risk (Blanco-Lopez et al., 2023; Muñoz-Aguirre et al., 2023) likewise concern disease epidemiology rather than nutritional treatment after diagnosis. Consequently, no specific diet can currently be recommended to alter leukemia control on microbiome grounds. Future trials should define nutritional status, fiber exposure, antibiotic use, fecal metagenomic function, circulating metabolites, and clinical endpoints, while avoiding restrictive diets that could worsen malnutrition during intensive therapy.

### 7.2 Fecal microbiota transplantation

FMT can reconstruct microbial communities more rapidly than diet or prebiotics, but its role in AL remains investigational. In AML models, FMT or selected bacteria reduced leukemic burden in association with restoration of butyrate-related barrier function (Wang et al., 2022). In patients with AML or allo-HSCT, oral or autologous microbiota products have shown feasibility, restoration of depleted anaerobes, reduction of *Enterococcus* expansion, and signals of lower infection risk or improved GVHD outcomes (Malard et al., 2021; Rashidi et al., 2023; DeFilipp et al., 2024; Youngster et al., 2024; Masetti et al., 2025). These studies differ in donor source, formulation, timing, conditioning, antibiotic exposure, and endpoint, and several include mixed transplant populations. The evidence therefore supports ecological restoration and early safety under controlled protocols, not a general claim that FMT improves leukemia response or survival. Rigorous donor screening, pathogen and antimicrobial-resistance surveillance, manufacturing standardization, and careful timing are essential in profoundly immunocompromised patients.

### 7.3 Probiotics and prebiotics

Probiotics and prebiotics offer less intensive forms of microbiota modulation, but their benefits and risks differ. Small studies in pediatric acute leukemia or HSCT suggest that *Lactobacillus rhamnosus*, *Lactobacillus* plantarum, synbiotics, fructo-oligosaccharides, resistant starch, or mixed fiber formulas may be feasible and may reduce selected gastrointestinal symptoms or mucosal complications (Ladas et al., 2016; Reyna-Figueroa et al., 2019; Yoshifuji et al., 2020; Andermann et al., 2021; Eghbali et al., 2023; Andersen et al., 2025). Representative probiotic and prebiotic studies in acute leukemia and related settings are summarized in **Table 2**. However, strain-level effects cannot be inferred from genus-level abundance, and a commensal associated with lower infection risk in one cohort should not automatically be labeled a predictive biomarker or administered as a probiotic (Šimiaková and Bielik, 2024; Wang H. et al., 2024). Viable organisms may translocate and cause bacteremia, sepsis, pneumonia, or endocarditis, particularly during neutropenia or severe mucosal injury (Guenther et al., 2010; Doern et al., 2014; Kothari et al., 2019; Lee et al., 2023; Merenstein et al., 2023). Prebiotics may avoid some risks of live organisms but can still produce variable metabolic effects. Future trials should specify strain or substrate, dose, timing, neutropenia status, mucosal integrity, antibiotic exposure, colonization, bloodstream surveillance, and a prespecified clinical endpoint.

**Table 2:** Summary of studies on probiotics and prebiotics in acute leukemia and related settings.

Intervention type	Representative study	Study population/setting	Intervention	Main findings
Probiotics	Reyna-Figueroa et al., 2019	Pediatric patients with acute leukemia receiving chemotherapy	Oral *Lactobacillus rhamnosus* supplementation	Reduced several chemotherapy-related gastrointestinal adverse effects, including nausea, vomiting, and abdominal distension.
Probiotics	Ladas et al., 2016	Children and adolescents undergoing hematopoietic stem cell transplantation	*Lactobacillus plantarum* supplementation	Demonstrated preliminary safety and feasibility.
Prebiotics	Andermann et al., 2021	Adults undergoing allogeneic hematopoietic stem cell transplantation	*Fructo-oligosaccharide* supplementation	Generally well tolerated, with no major safety concerns.
Prebiotics	Andersen et al., 2025	Patients after allogeneic hematopoietic stem cell transplantation	Enteral prebiotic fiber supplementation	Generally well tolerated; may increase beneficial bacteria and reduce microbiome pathways related to antimicrobial resistance.
Prebiotics	Yoshifuji et al., 2020	Patients after allogeneic hematopoietic stem cell transplantation	Resistant starch and a glutamine/fiber/oligosaccharide formula	Mitigated mucosal injury and reduced the incidence of overall acute GVHD and grade 2–4 acute GVHD.
Synbiotics	Eghbali et al., 2023	Children with ALL receiving maintenance chemotherapy	LactoCare synbiotic	Reduced chemotherapy-induced diarrhea and improved gastrointestinal tolerance.

## 8 Limitations

This review has several limitations. First, the AL literature is dominated by small observational cohorts, and direct causal evidence in human disease is scarce. Second, study populations differ substantially in age, AML versus ALL subtype, disease status, treatment phase, antibiotic exposure, diet, geographic setting, and transplant context. Third, sequencing platforms and bioinformatic pipelines are heterogeneous, and taxonomic composition is often reported without measurement of microbial function or systemic metabolite exposure. Fourth, many mechanisms discussed for DCs, T cells, B cells, macrophages, NK cells, and hematopoietic progenitors derive from non-leukemia models and are explicitly presented as contextual evidence rather than AL-specific proof. Fifth, positive intervention studies may be preferentially published, while negative ecological or clinical results are less visible. Finally, the proposed two-hit and gut-bone marrow models are integrative hypotheses that require experimental validation.

These limitations also constrain interpretation of contradictory taxa. A microbial change observed after induction therapy may reflect chemotherapy, antibiotics, mucositis, diet, or hospitalization rather than leukemia biology. Conversely, similar clinical phenotypes may arise from different taxa that share the same metabolic function. Future studies should therefore prioritize longitudinal within-patient designs, standardized metadata, shotgun metagenomics or metatranscriptomics, targeted and untargeted metabolomics, barrier markers, immune phenotyping, and paired bone marrow analysis. Claims of causation should require temporal precedence, dose-response or perturbation evidence, pathway-specific blockade, and rescue.

## 9 Conclusions and future perspectives

Current evidence supports a close but incompletely resolved relationship among gut dysbiosis, barrier injury, systemic inflammation, immune-cell remodeling, treatment exposure, and clinical outcomes in AL. The most reproducible human findings concern treatment-related ecological disruption, infection risk, and allo-HSCT outcomes. AL-specific mechanistic evidence is narrower, with the clearest example linking reduced butyrate-related barrier support to LPS leakage and accelerated AML progression in mice (Wang et al., 2022). It remains unproven that microbiota-derived metabolites directly reprogram human leukemia blasts or that microbiota-directed interventions improve leukemia control. The evidence-graded gut-bone marrow framework proposed here shifts the field from lists of taxa toward defined routes, target cells, receptors, and endpoints. Progress will depend on paired longitudinal sampling, functional multi-omics, mechanistic perturbation, and intervention trials designed around the patient’s disease subtype and treatment context. Until such evidence is available, microbiota-based strategies should be viewed as investigational supportive approaches rather than established components of precision leukemia therapy.

## 10 Research priorities

Five priorities follow from this framework. First, determine whether dysbiosis precedes or follows leukemia and treatment by sampling patients before antibiotics and longitudinally through induction, remission, relapse, and transplantation. Second, identify the route to marrow by measuring matched stool function, plasma microbial products and metabolites, and bone marrow exposure, supplemented by isotope tracing or microbial barcoding in experimental models. Third, define the target cell by testing receptor-dependent responses in stromal cells, macrophages, hematopoietic progenitors, immune cells, and genetically characterized leukemia blasts. Fourth, test immune-cell trafficking with paired gut, blood, and marrow clonotypes and chemokine-receptor perturbation. Fifth, conduct biomarker-guided intervention trials in which ecological restoration, infection, toxicity, immune recovery, remission, and survival are prespecified as separate endpoints. These studies can determine which parts of the gut-bone marrow model are causal, clinically useful, or merely correlated.
